# Failure of Glass Fibre-Reinforced Polypropylene Metal Laminate Subjected to Close-Range Explosion

**DOI:** 10.3390/polym12092139

**Published:** 2020-09-19

**Authors:** Amin Bassiri Nia, Li Xin, Mohd Yazid Yahya, Amran Ayob, Ali Farokhi Nejad, Seyed Saeid Rahimian Koloor, Michal Petrů

**Affiliations:** 1 Faculty of Engineering, School of Mechanical engineering, University Technology Malaysia, Skudai 81310, Malaysia; amin.bassirinia@gmail.com (A.B.N.); amranayo@gmail.com (A.A.); ali.farokhi@polito.it (A.F.N.); 2 National Laboratory of Solid State Microstructures & Department of Material Science and Engineering, Nanjing University, Nanjing 210093, China; lixin1986@tyut.edu.cn; 3 Department of Mechanical and Aerospace engineering, Politecnico di Torino, 10129 Torino, Italy; 4 Institute for Nanomaterials, Advanced Technologies and Innovation (CXI), Technical University of Liberec (TUL), Studentska 2, 46117 Liberec, Czech Republic; michalpetru@tul.cz; 5 Department of Aerospace Engineering, Faculty of Engineering, Universiti Putra Malaysia, Serdang 43400, Malaysia

**Keywords:** composite, hybrid structure, blast, impulse, failure mode and deformation

## Abstract

The present study investigates the effects of close-range blast loading of fibre metal laminates (FMLs) fabricated from woven glass polypropylene and aluminium alloy 2024-T3. The polypropylene layers and anodized aluminium are stacked in 3/2 layering configuration to investigate the impact energy absorbed through deformation and damage. In order to study the blast responses of FMLs, a 4-cable instrumented pendulum blast set-up is used. Effects of blast impulse and stand-off distance were examined. Investigation of the cross-section of FMLs are presented and damages such as fibre fracture, debonding, and global deformation are examined. Increasing stand-off distance from 4 to 14 mm resulted in a change of damage mode from highly localized perforation to global deformation.

## 1. Introduction

**Figure 1:**
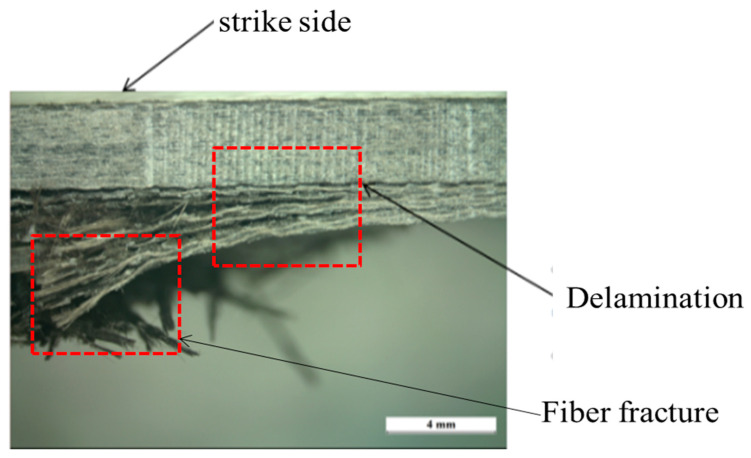
Cross-section of basalt-epoxy laminate after being subjected to 40 g TNT and 200 mm SOD [9].

Fibre-reinforced polymer (FRP) laminates, fibre metal laminates, and sandwich composites are used in a broad range of light-weight structures such as armoured vehicles, aerial drones, tanks, ships, and aircraft. Accordingly, the investigation of FRP is highlighted when the assets at risk of attack using explosive munitions. Many researchers studied the effect of fibre material in FRP laminate [1], resin material in laminate [2] and geometry of laminate [3,4] under impulsive loading. In the case of fibre effect on blast resistance, Tekalur et al. have conducted a research which has indicated that failure in carbon-reinforced laminate is dominated by a sudden destructive brittle form of damage [1]. In contrast, the laminate with E-glass fibre reinforcement demonstrates progressive damage under impulsive loading. In the case of matrix effect on blast resistance, Yahya and his colleagues have indicated epoxy resin does not improve the blast resistance in comparison with thermoplastic resin [2]. The blast response of carbon/epoxy FRP has been studied by Yahya et al. Their research has presented that the delamination and fibre fracture are the dominant damage in the laminate when exposed to explosion [4]. When metal is exposed to impulsive loading most of the blast energy is dissipated during considerable plastic deformation [5,6]. However, when exposed to similar loading, fibre-based polymer usually tends to fail in the form of fibre fracture and matrix cracking [7]. Chai and Manikandan [8] have proven that the fibre-based polymer has a low inter-laminar shear strength and tensile strength subjected impulsive loading, which causes the delamination and fibre fracture in fibre-based polymer laminate (Figure 1). To overcome this issue, using metal in the laminate can reinforce the structure as fibre metal laminates (FML), especially in transverse loading conditions [8].

Hybrid FMLs have positive characteristics such as high strength [10], impact resistance, fire resistance, corrosion resistance [11], energy absorption [12,13], and weight saving [14]. These characterizations make them a strong candidate for different applications such as aerospace and automotive structures. In recent years, there are some well-known FMLs have been introduced such as the laminate of aluminium reinforced with glass fibre (GLARE), the laminate of aluminium reinforced with carbon fibre (CARAL), and the laminate of aluminium reinforced with aramid fibre (ARALL). GLARE is more useful in aeronautic industries application [15,16]. Previous studies have compared these three FMLs by impact tests to evaluate the back face deflection and energy absorption. The highest impact resistance was achieved by GLARE in comparison to ARALL and CARAL [17]. The fabrication of GLARE panels using thermoset resin is time-consuming [18], often resulting in high fabrication cost and reduced productivity. To overcome this problem, the alternative use of thermoplastic resin offers less curing time [14]. Based on previous research [19] it can be said that the manufacturing process is the main issue in terms of final price of FML. However, to obtain a lightweight and stiff structures the price of FML is reasonable in compare with conventional metal or FRP composites [19]. Furthermore, by comparing different thermoplastic resin such as polypropylene, high density polyethylene, low density polyethylene, nylon 6.6, and nylon 6, the lowest density polypropylene can reach 700% elongation at breakage [18] which is useful in dissipating the blast energy. Some researchers investigated fibre metal laminates [20,21,22,23] or sandwich structure with FML face sheet [24] over impulsive loading through experimental or FEM method [25,26]. Langdon et al. have defined the blast pressure distribution as a function of time and radial position of FMLs experimentally [27]. Some studies were carried out to investigate the effect of blast loading on FMLs with aluminium face sheet and glass fibre-reinforced polypropylene (GFPP) laminate in fixed stand-off distance [28,29]. The strain rate dependency of the glass fibre polypropylene laminate was experimentally proved by Brown et al. [30]. Furthermore, this characterization was used to define strength rate dependent of candidate material GFPP in FMLs modelling during blast loading [25,26,27,28,29]. Different damage models have been used to calculate the effect of GFPP damage in FMLs modelling when exposed to localized loading. Several studies have been conducted to use instant damage failure model and damage variables to control the degradation of the stiffness matrix, hence defining the damage evolution in composite material response [25,26,29]. In FML structures the stress wave propagation through thickness has a significant effect in the final damage mode and panel response. By changing the number of layers (different number of interfaces) and different layer arrangements and changing material properties through-thickness, the stress wave propagation is changed. These results in different panel response and final damage [20]. The stress propagation and damage of FML in impulsive loading are also related to fibre orientation in the composite [20,21]. Several experimental studies have been carried out on the effect of impulsive energy quantitatively [20,31]. They perform a test on a laminate with 11 layers and increased the impulse energy from 4.2 to 14 N.s. The maximum back face deflections were observed to increase from 6 to 32 mm. The results also indicated that for panels with 28 layers (thick FMLs), by amplification of impulse from 12 to 28 N.s, the maximum back face deflection increases from 22 to 30 mm [31]. The blast energy is mostly dissipated by different damage mechanisms such as fibre fracture, fibre stretching and plastic deformation in FMLs during localized explosion [32] and debonding does not show a significant role in dissipating the blast energy [32].

In this paper, the effect of blast intensity and pressure distribution is investigated on FML panels with 3/2 lay-up configuration for response and failure mechanism. The blast intensity is increased by increasing the weight of spherical shaped explosive and the pressure distribution is changed by varying the stand-off distance in the close-range explosion between 4 and 14 mm. Failure mode I is referred to the back face large inelastic deformation, which will be refined based on the back face damage shape. The back face aluminium tearing is defined as failure mode II.

## 2. Panel Fabrication

**Figure 2:**
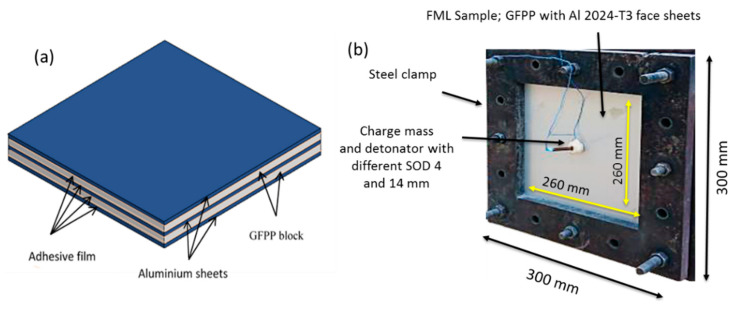
(**a**) The schematic showing the lay-up configuration, (**b**) dimension of the exposed area for a fibre metal laminates (FML) sample.

The FML panels are fabricated from 3 layers of aluminium sheet grade 2024-T3 with thickness 1 mm and woven E-glass reinforced polypropylene composite. The nominal specific weight is 745 g/m^2^ and the 8 layers of GRPP composite have a fibre weight fraction of 60%. The panel was fabricated 300 mm × 300 mm in size, and the total thickness of the aluminium sheets and GFPP layers is kept fixed. Since the polypropylene matrix exhibits low surface energy characterization and low bonding quality, the aluminium sheet was anodized and adhesive film Xiro 23.101 (Collano AG, Neulandstrasse, Switzerland) was placed between GFPP laminate and aluminium sheet during fabrication. The desired layering arrangement of 3/2 was prepared in the mould, then it was heated in the oven at 190 °C and finally was cold pressed at 16 bars. The schematic of the FML is shown in Figure 2a. Each GFPP block, with mean thickness 2 mm, contain 4 layers of woven E-glass reinforced polypropylene. An adhesive film with thickness 60 μm is placed between aluminium and GFPP blocks. The thickness and weight of the FML panels vary by ±2% and ±1%, respectively. The mean metal volume fraction in the panels is 42%. The lay-up arrangement, weight, and thickness of the samples are shown in Table 1.

## 3. Experimental Procedure

**Figure 3:**
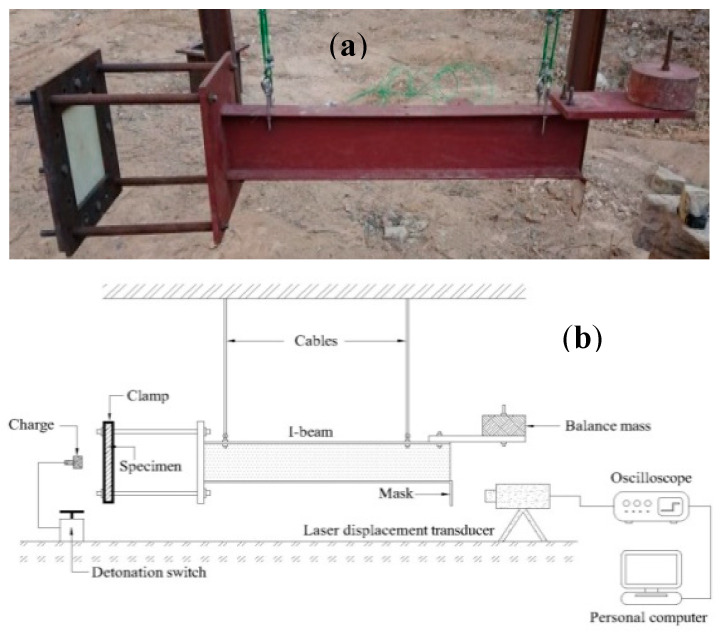
The blast test set up (**a**) four-cable pendulum blast system. (**b**) Schematic of the four-cable pendulum blast set up.

The blast load was generated by detonating the emulsion explosive, which is spherical in shape, resulting in an impulse velocity of 5200 m/s. The four-cable pendulum blast system (Figure 3) was used to measure the blast intensity. The FML panel was clamped in front of the ballistic pendulum, exposing an area 260 mm × 260 mm facing the charge (see Figure 2b). The explosive mass was varied from 10 to 30 g and was placed at stand-off distance of 4 and 14 mm from the centre of the panel. This setup is designed to provide different blast pressure distribution on the exposed area of FML panel [33]. The impulse is calculated from the oscillating movement of the pendulum after the explosion. The movement of the pendulum was recorded using laser Micro-Epsilon LD1607-200 (Micro-Epsilon Messtechnik, Ortenburg, Germany).

Furthermore, the motion formula for ballistic pendulum [34] is used to calculate the impulse from detonation. The general solution of the equation is given by Equation (1) and the two specific solutions in time T4 and 3T4 are given by Equations (2) and (3), respectively.

**Figure 4:**
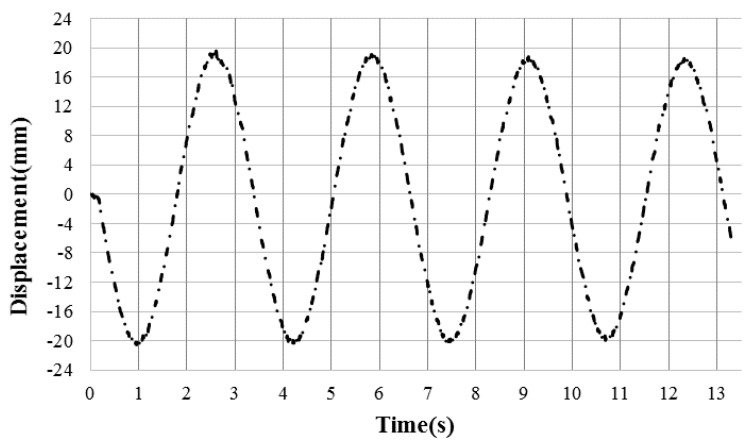
Pendulum motion data recorded by laser for 10 g spherical emulsion explosive in 4 mm standoff distance.

(1) X = e−βt x.°ω sin(ωt) 

(2) X1=e−βT4x0˙T2π

(3) X2=e−3βT4x0˙ T2π

and finally, the input impulse on the structure can be calculated by the following expression:

(4) I=MTx0˙=MTx12 πTeβT4  and  β= 2lnx1x2T

where $M_{T\;}$ = 165 kg, is the total mass of pendulum and *T* = 3.24 s refer to the time for one cycle of oscillating motion. For illustration, Figure 4 shows the recorded pendulum motion due to the detonation of 10 g of spherical emulsion explosive in 4 mm stand-off distance from the panel. Here $x_{1}$ = 20.538 mm, $x_{2}$ = 19.6635 mm, β = 0.0268, *T* = 3.24 s, $M_{T\;}$ = 165 kg. Therefore, from Equation (4) the impulse (I) is 6.7 N.s.

The back face deformation from non-perforated post-blast test FML samples was recorded using the Coordinate Measuring Machine (ZEISS, Oberkochen, Germany). The machine measures the physical geometry of the deformation when the probe is moved in the perpendicular direction through the centreline of the sample. The samples are grouped into group A and group B, referring to samples in stand-off distances of 4 and 14 mm, respectively. The stand-off distance, weight of explosive, the calculated impulse for the corresponding mass of explosive, the maximum back face deflection, and the failure mode are listed in Table 2.

## 4. Results and Discussions

### 4.1. Failure Mode Definition

According to Langdon et al.’s study [21] the failure modes are categorized. Based on the back face damage observation, different failure modes are reported in Table 2. From the close-range blast test, different damage on the back face, such as global deformation and metal tearing were observed and are grouped as failure modes I and II, respectively [20]. Some experimental work focusing on failure mode I have been carried out. They observed the diamond shaped debonding and hexagonal shaped debonding [20,21]. A circular nose shaped damage was also observed at the centre of the back face in experimental work by [20]. For mode II failure, back face metal tearing or petalling was observed for thin FMLs in higher intensity impulse [20,21]. In the present study, the back face metal can be damaged in the form of circular nose (I-CN), circular flat shape (I-CF), hexagon shape (I-H), or diamond shape (I-D). In failure mode I, the back face damage forms the ultimate failure of the panel, for example, the sample code S14141-4 finally failed with hexagon back face damage I-H. It is worth mentioning that debonding causes a damage with a particular shape on the back face of panels however, this damage is different from the damage grouping in this study. For example, the sample code S14141-6 shows the diamond shaped damage from debonding, but the back face metal damage form for this sample is circular nose-shaped, and the failure type for this sample is represented by mode I–CN. 

### 4.2. Response of FMLs in Group A

**Figure 5:**
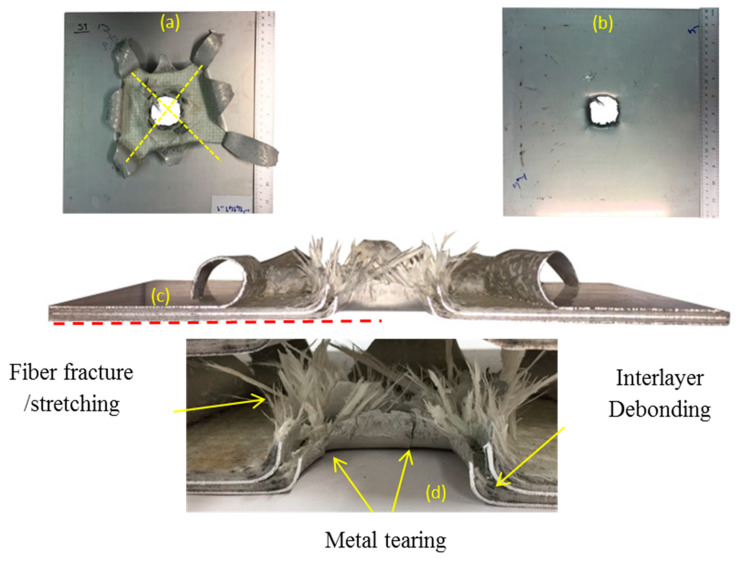
Sample code S14141-1 in impulse 16.5 N.s (**a**) back face, (**b**) front face, (**c**) cross section, (**d**) close-up view of cross section.

Detonation using 10, 15, and 20 g of spherical shaped emulsion explosive at 4 mm stand-off distance from the panels generated impulses of 6.7, 12.5, and 16.5 N.s, respectively. The maximum central back face deflection, the global deformation, failure mode and damage type of glass-based metal laminate in this group are discussed. The samples in group A were exposed to impulse of 16.5 N.s, resulting in huge damage. The pictures of the back face, exposed face, and the cross-section of the panel are shown in Figure 5a–c, respectively. From an explosion of 20 g emulsion at 4 mm SOD, the panel was perforated with 50 mm diameter hole at the centre and experienced extensive metal petalling and fibre fracture. The back face metal tearing is classified as mode II failure, which occurs at high impulsive loading and small stand-off distance. It is obvious that for through thickness perforation to occur the front face should also experience metal tearing damage. The global deformation of the front face was insignificant. The front and internal aluminium sheets show backward petalling (Figure 5d), and for back face aluminium sheet the petals expanded outward and preferably in the diagonal direction.

**Figure 6:**
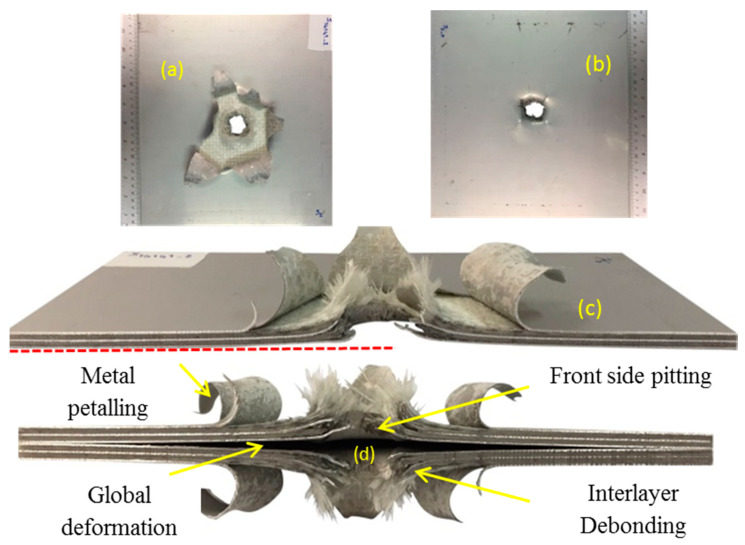
Sample code S14141-2 in impulse 12.5 N.s (**a**) rear face, (**b**) front face, (**c**) cross section, (**d**) double side cross section.

The sample also demonstrates a small amount of debonding for the front and internal aluminium sheets and high debonding length for the back aluminium sheet. The impulse 12.5 N.s is the result of detonating 15 g spherical-shaped emulsion explosive at 4 mm stand-off distance. Figure 6a–c represents rear face sheet, front face sheet, and cross-section of S14141-2, respectively. Failure of the sample is dominated by mode II, which shows a perforated sample with 25 mm diameter hole at the centre of the panel. Figure 6d shows a cross-section which includes both sides of the selected sample. Different types of damage such as global deformation, pitting of the front face, fibre fracture, debonding, and back face petal are identified. The front face shows metal tearing and global deformation for this magnitude of impulse. At the perforation, the front and internal aluminium layers petal backwards while the back face sheet tulip outward. Detonating a 10 g spherical emulsion 4 mm from the panel generates an impulse 6.7 N.s.

**Figure 7:**
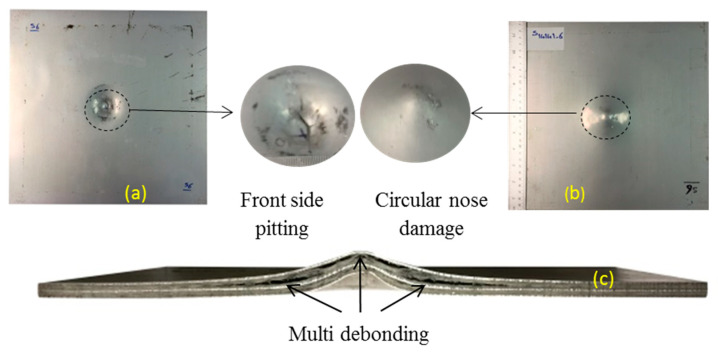
Sample S14141-6 in impulse 6.7 N.s (**a**) front face, (**b**) back face, (**c**) cross section.

The pictures of the front face, rear face sheets, and cross-section of selected samples of this group are shown in Figure 7a–c, respectively. The front face of the selected sample experienced metal tearing and pitting. Debonding of internal layers and back face sheet are observed. The diamond-shaped debonding damage is visible for the back face. The back face metal is damaged in circular nose shape. These samples are classified as failure mode I-CN.

### 4.3. Response of FMLs in Group B

**Figure 8:**
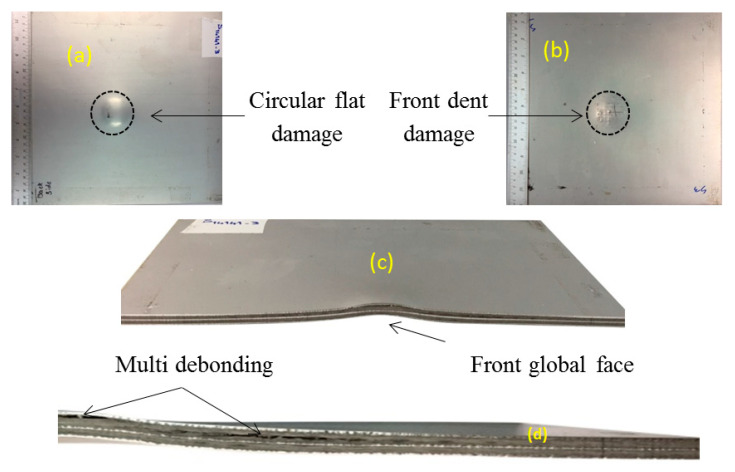
Sample code S14141-3 subjected to impulse 14.7 N.s (**a**) back face, (**b**) front face, (**c**) cross-section, (**d**) half cross-section.

Close-range explosion tests on FMLs have been considered by researchers [20,21,25]. For example, the localized blast loading on FMLs was imposed by detonating the disk shape PE4 explosive in SOD of 14 [20,21] and 13 mm [35] and uniform blast loading at SOD of 13 mm was performed by [22]. Samples with codes S4141-3, S14141-4, and S14141-5 were subjected to blast loading from the detonation of 15, 20, and 30 g spherical emulsion explosives at 14 mm SOD. The recorded impulses from the detonation are 14.7, 18.8, and 31.2 N.s, respectively. The first sample in this group, with code S1414-3, experienced impulse 14.7 N.s from the detonation of 15 g explosive. The photos of the back face, front face, and cross-section of the sample are shown in Figure 8a–c, respectively.

The global deformation and the pattern of woven appear on the front aluminium face sheet and the back face was damaged in a circular shape. A comparison of explosive with different shapes has been carried out previously [36]. In that work the authors have proven that, regarding a constant SOD, the peak pressure of 50.16 g spherical shape would be equal to 40 g explosive in cylindrical shape. Debonding appears on the internal and back face sheet, which is longer in length than in the middle aluminium layer. The maximum back face deflection for this sample reaches 7.9 mm. The overall failure for this sample is defined as mode I-CF. The sample does not show any metal tearing damage, fibre fractures or delamination. 

**Figure 9:**
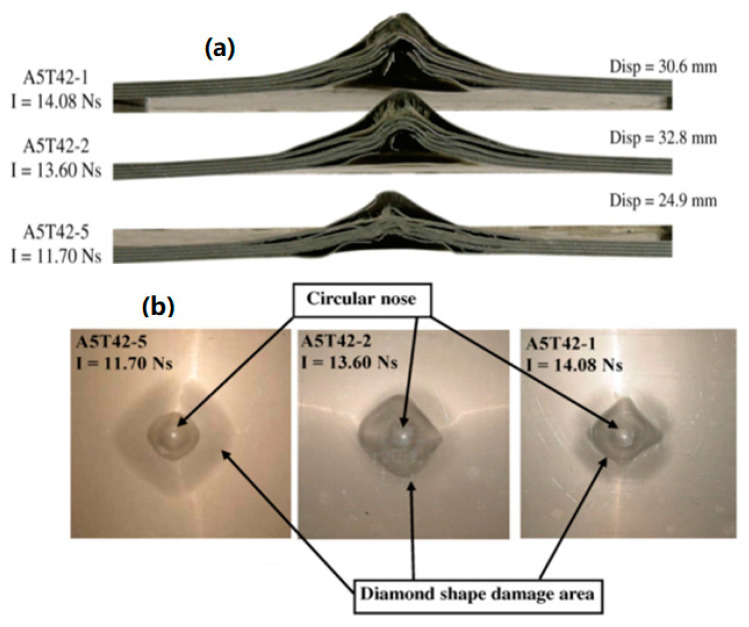
(**a**) Cross-section of sample in Ref. [31] (**b**) back face damage in Ref. [20].

The results for these samples become more important when compared with samples with code A5T42-# from [20]. In the mentioned article [20], the glass-based polypropylene metal laminate using 5 layers of aluminium has been fabricated. Their samples consist of 8 layers of GFPP with a thickness of 0.6 mm. The resulting 7.4 mm thick FML panel contains aluminium with 43% volume fraction. When exposed to impulse between 11.70 and 14.08 N.s the back face debonding length increases (Figure 9a) and a circular nose shape damage appears on the back face (Figure 9b). By comparing the results for sample code S1414-3 and samples with code A5T42-# from [20] it can be concluded the disk shape explosive causes more localized damage than spherical shape explosive and the distribution of the metal sheets in the panel affect damage. The final failure modes are different by using 3 layers of 1 mm thick aluminium sheets compared to 5 layers of 0.6 mm thick aluminium sheets. Samples A542-# from Ref [20] have more interfaces and the blast waves have to travel through more thickness interfaces. This causes higher reflected blast pressure that causes more debonding in the panel and different back face failure mode.

**Figure 10:**
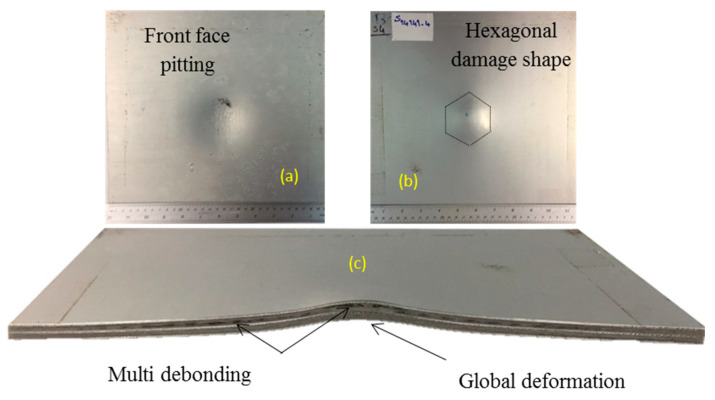
Sample code S14141-4 subjected to impulse 18.8 N.s (**a**) front face, (**b**) back face, (**c**) cross-section.

The second sample in this group (sample code S14141-4) was subjected to impulse 18.8 N.s from the detonation of 20 g spherical explosives at 14 mm stand-off distance. The photographs of the exposed face, non-exposed face and cross-section are shown in Figure 10a–c, respectively. The front face experiences high global deformation. The woven pattern of the glass fibres is also apparent on the front face of this sample.

**Figure 11:**
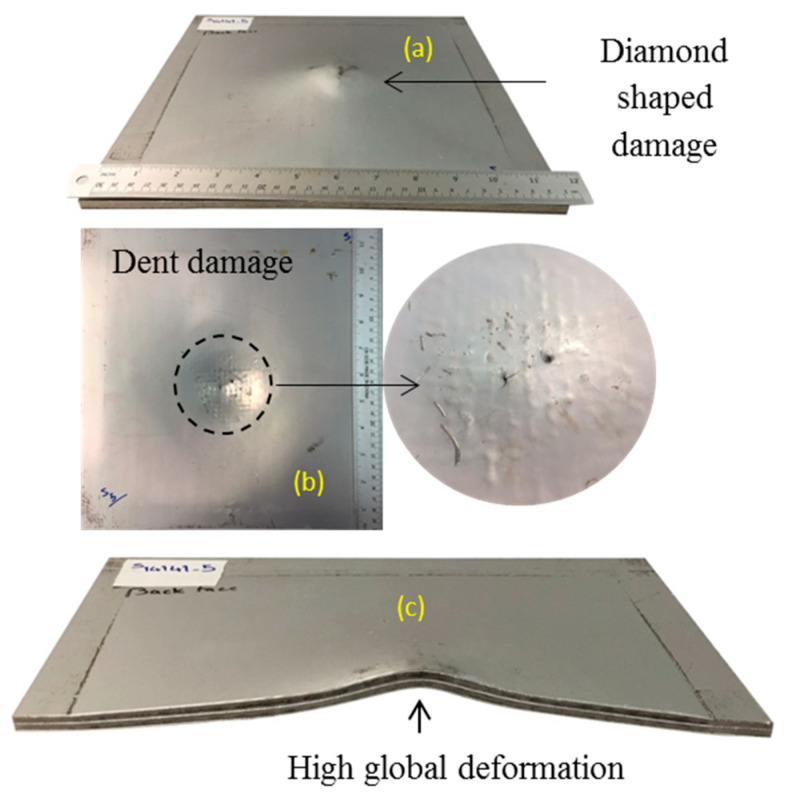
Sample code S14141-5 subjected to impulse 31.2 N.s (**a**) back face, (**b**) front face, (**c**) cross-section.

**Figure 12:**
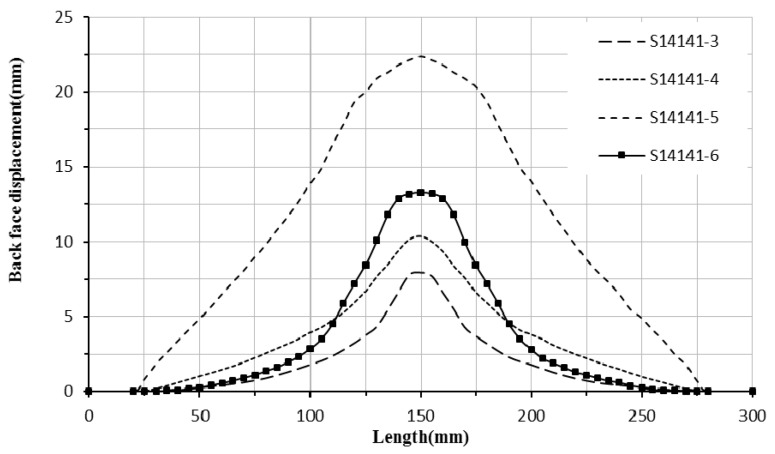
The back face deformation for non-perforated samples (i.e., general failure mode I).

The sample code S14141-5 was subjected to the blast pressure from detonation of 30 g spherical shaped emulsion explosive at 14 mm stand-off distance. The photographs of the back face, front face, and cross-section are shown in Figure 11a–c, respectively. The pattern of woven fibre is evident on the front face of the sample. Diamond-shaped debonding and back face diamond-shaped damage dents are visible. The debonding for internal aluminium layers extends to the edges of the sample. This sample experiences high global deformation of the front and back face sheets with maximum 22.36 mm deflection of the centre of the back face. The back face global deformation for this sample is shown in Figure 12. The sample is considered to have failed in mode I-D, which means the back face experiences global deformation (not metal tearing) with diamond-shaped back face damage. The back face and internal aluminium sheets experience detachment from the GFPP composite block due to debonding. The back face metal sheet is damaged with hexagon shape indentation and hence is grouped as failure mode I-H. The global deformation of the back face sheet for the sample is shown in Figure 12. 

### 4.4. Behaviour of FML Panel against Blast Impulse

For FML samples in groups A and B by increasing the impulse, the damage intensity increases and failure mode changes. In group A, by increasing the intensity of impulse, the front face and internal layer debonding lengths decrease, and rear face debonding length increases in size. These are due to localized blast wave propagation at the nearer stand-off distance of 4 mm. For samples in group A which demonstrate failure mode II, the blast energy is dissipated by panel perforation. The number of tulips and size of tulips also increase by increasing the impulse intensity.

For group B samples, by increasing the impulse from 14.7 to 31.2 N.s, the maximum deflection at the centre of the panel increases from 7.9 to 22.36 mm, that is, the global deformation increases by increasing the impulse. By increasing the impulse, the rear face damage dent changes from circular shape to diamond shape. The internal debonding length increases and extends to the boundary (panel edges) in high impulse loading. When subjected to higher stand-off distance the samples are exposed to a larger area of the blast loading. The blast energy is mostly absorbed by panel global deformation. Hence, increasing the impulse intensity leads to an increase of the global deformation.

### 4.5. The Effect of Stand-Off Distance in Close-Range Explosion

The stand-off distance has a significant role in blast wave propagation for far-range explosion. Researchers have indicated by increasing the SOD, the pressure arrival time, and pressure time increase, the peak reflected pressure decreases and degradation of pressure-time decreases [7,37]. In this experimental investigation, by increasing the SOD from 4 to 14 mm, the reflected load pressure at the centre of the front face decreases and degradation of pressure-time decreases. A larger area of the panel is affected by the blast and this in turn causes global deformation instead of panel perforation. Comparing the test results from groups A and B it can be concluded the pressure distribution and the resulting damage of the panel are highly sensitive to close field and stand-off distance. With the same mass of explosive (15 and 20 g) and increasing the SOD by 10 mm in close-range test (4 to 14 mm), the general form of failure mode changes from mode II to mode I. Comparing the test results, panels having mode I failure show significant effect of close-range SOD between groups A and B. It is observed by increasing the SOD by 250% in close-range test (4 to 14 mm) and increasing the impulse by 120% and 180% the maximum back face displacements are reduced by 40% and 22%, respectively. The sample code S14141-5 demonstrates that by increasing the impulse and SOD in close-range test by 365% and 250%, the back face deflection is increased by 68%. The panel does not suffer any metal tearing or fibre fracture and the blast energy is dissipated by global panel deformation and internal debonding rather than metal tearing and panel perforation. This can be explained by the impulse loading maintaining higher time-pressure and lower reflected pressure.

## 5. Conclusions

The effect of impulse intensity and SOD in close-range blast on GFPP FML is summarized in the following:

For 4 mm SOD, (FML in group A) by promoting the impulse from 6.7 to 16.5 N.s, the back face sheet debonding length and number of tulips increase. However, the internal debonding length and front face global deformation are diminished. By increasing the impulse, the general form of damage changes from global deformation to localized perforation. For 14 mm SOD (FML in group B), by promoting the impulse from 14.7 to 31.2 N.s, the global deformation dominates other types of damage. The internal debonding length and global deformation increase. Increasing the SOD by 250% (10 mm), the localized damage changes to global deformation. Hence, small SOD has a significant role in material response and damage form, which should be considered in FML structure design. 

## Figures and Tables

**Table 1:** Samples lay-up arrangement, thickness, and weight.

Samples Code	Lay-Up Arrangement FML (3/2)	Thickness (mm)	Weight (g)
S14141-1	1/4/1/4/1	7.11	1160
S14141-2		7.09	1165
S14141-6		7.13	1154
S14141-3		7.09	1161
S14141-4		6.98	1168
S14141-5		7.1	1152

**Table 2:** Stand-off distance, weight of explosive, the calculated impulse for the corresponding mass of explosive, the maximum back face deflection, and the failure mode.

Group	FML Code	Mass of Charge (g)	SOD (mm)	Impulse (N.s)	Failure Mode	Backface Deflection (mm)
A	S14141-1	20	4	16.5	II	----
	S14141-2	15	4	12.5	II	----
	S14141-6	10	4	6.7	I-CN	13.31
B	S14141-3	15	14	14.7	I-CF	7.9
	S14141-4	20	14	18.8	I-H	10.36
	S14141-5	30	14	31.2	I-D	22.36
